# Sub-Regional Differences of the Human Amniotic Membrane and Their Potential Impact on Tissue Regeneration Application

**DOI:** 10.3389/fbioe.2020.613804

**Published:** 2021-01-13

**Authors:** Adelheid Weidinger, Laura Poženel, Susanne Wolbank, Asmita Banerjee

**Affiliations:** ^1^Ludwig Boltzmann Institute for Experimental and Clinical Traumatology, AUVA Research Center, Vienna, Austria; ^2^Austrian Cluster for Tissue Regeneration, Austria

**Keywords:** human amnion, human amniotic membrane mesenchymal stromal cells, human amniotic membrane epithelial cells, sub-regions, placental, reflected, scaffold, bioactive factors

## Abstract

For more than 100 years, the human amniotic membrane (hAM) has been used in multiple tissue regeneration applications. The hAM consists of cells with stem cell characteristics and a rich layer of extracellular matrix. Undoubtedly, the hAM with viable cells has remarkable properties such as the differentiation potential into all three germ layers, immuno-modulatory, and anti-fibrotic properties. At first sight, the hAM seems to be one structural entity. However, by integrating its anatomical location, the hAM can be divided into placental, reflected, and umbilical amniotic membrane. Recent studies show that cells of these amniotic sub-regions differ considerably in their properties such as morphology, structure, and content/release of certain bioactive factors. The aim of this review is to summarize these findings and discuss the relevance of these different properties for tissue regeneration. In summary, reflected amnion seems to be more immuno-modulatory and could have a higher reprogramming efficiency, whereas placental amnion seems to be pro-inflammatory, pro-angiogenic, with higher proliferation and differentiation capacity (e.g., chondrogenic and osteogenic), and could be more suitable for certain graft constructions. Therefore, we suggest that the respective hAM sub-region should be selected in consideration of its desired outcome. This will help to optimize and fine-tune the clinical application of the hAM.

## Introduction

The human amniotic membrane (hAM), a tissue of embryonic origin that encloses the fetus and the amniotic fluid, providing nourishment and protection, has been used for tissue regeneration for over a century [reviewed in Silini et al. (2015)]. First described by Davis (1910), it has been used as wound dressing material (Andonovska et al., 2008), and in reconstructive surgery (Brindeau, 1934; Nisolle and Donnez, 1992), particularly in ophthalmology (De Rötth, 1940; Seitz et al., 2009). The hAM has served as a decellularized natural biomaterial and a potential source for cells for numerous clinical applications. After decellularization, the hAM still consists of a rich layer of extracellular matrix, including specific proteins and bioactive substances, favored by cells in general.

In recent years, however, research has turned toward properties of viable amniotic cells. The hAM harbors two distinct cell populations, the human amniotic membrane epithelial cells (hAECs) and the human amniotic membrane mesenchymal stromal cells (hAMSCs). Both cell types show characteristics that can be attributed to stem cells. This includes the expression of pluripotency markers (Miki et al., 2007) and mesenchymal stem cell markers (In’t Anker et al., 2004; Portmann-Lanz et al., 2006). The two cell populations hAECs and hAMSCs differ in their marker expressions, and have been described elsewhere (Parolini et al., 2008). Furthermore, hAECs and hAMSCs have the potential to differentiate toward cell lineages of all three germ layers *in vitro* and *in vivo* (Sakuragawa et al., 1996, 2000; Kakishita et al., 2003; In’t Anker et al., 2004; Portmann-Lanz et al., 2006). In addition, the hAM and amniotic cells have anti-inflammatory (Bailo et al., 2004; Wolbank et al., 2007) and immune-modulatory (Wolbank et al., 2007; Kronsteiner et al., 2011) properties, and tissue rejection upon transplantation does not occur. No reports exist of tumorigenic conversion of the cells, and although of embryonic origin, utilization of the hAM does not raise any ethical concerns. But there is more to it. What, at first sight, seems to be one structural entity, at a closer look, considering its anatomical site, turns out to be a multi-facetted, highly organized system. *In utero*, the hAM covers the placenta (placental amnion), and it lines the uterine wall (reflected amnion), and the umbilical cord (umbilical amnion) (Benirschke et al., 2006). Several studies have shown that cells and cell organelles of these amniotic regions differ considerably in their properties. In the past, these regional differences of the hAM were especially taken into account to investigate mechanisms that contribute to preterm or term rupture of the membranes. However, regional differences of the hAM have not yet been considered when selecting grafting material for clinical application. Since the hAM acts at extra- (e.g., attraction of cells) and intracellular levels (e.g., induction of differentiation) for tissue regeneration, it can be assumed that different cellular properties, owed to different amniotic regions, would impact tissue regeneration to a certain extent. The aim of this review is to summarize findings regarding different properties of the sub-regions of the hAM and to discuss their possible relevance for tissue regeneration. As for the application for tissue regeneration predominantly term placentae of cesarean sections are used, we focused solely on these.

## Sub-Regions of the Human Amniotic Membrane

**FIGURE 1:**
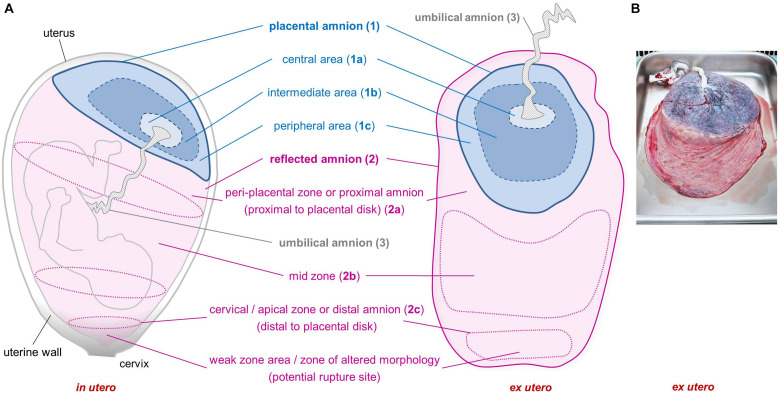
Sub-regions of the human amniotic membrane. Schematic of placenta and amniotic membrane *in utero* and *ex utero* **(A)**. Image of placenta and fetal membranes before preparation of amniotic membrane **(B)**. Placental amnion (1) (blue; covers the placenta), reflected amnion (2) (pink; lines the uterine wall), and umbilical amnion (3) (gray; covers the umbilical cord). Placental amnion (1) can be divided into a central area (1a), which is closest to the umbilical cord; an intermediate area (1b); and a peripheral area (1c) at the edge of the placental disk. Reflected amnion (2) is further divided into a peri-placental zone or proximal amnion (2a), which is the area closest to the placental disk; a mid-zone (2b); and a cervical/apical zone or distal amnion (2c), the area farthest from the placental disk. The weak zone (or zone of altered morphology) is located in the cervical zone and is the zone where the rupture of the membranes occurs before the onset of labor.

Emge (1921) already recommended to consider different sub-regions of the hAM, as he observed morphological differences in cells within the amniotic membrane. According to the anatomical site, the hAM can be divided into different sub-regions (Figure 1). The placental amnion [Figure 1A (1)] covers the placenta, and the reflected amnion [Figure 1A (2)] lines the uterine wall, thereby forming the amniotic sac. The amnion covering the umbilical cord [Figure 1A (3)] is termed umbilical amnion or cord amnion (Benirschke et al., 2006).

In these amniotic sub-regions, epithelial cells differ distinctly in their morphological appearance. The epithelium of the reflected amnion consists of a single layer of cuboidal cells with central nuclei whereas epithelial cells of the placental amnion are cylindrical with decentralized apical nuclei (Thomas, 1965; van Herendael et al., 1978; Han et al., 2008; Yoon et al., 2014; Banerjee et al., 2015; Deihim et al., 2016; Lemke et al., 2018). In contrast, the umbilical amnion consists of three to four layers of epithelial cells (van Herendael et al., 1978; Yoon et al., 2014). Perinuclear halos can be seen in umbilical amnion cells of the lower layers whereas cells of the upper layers show pyknotic nuclei or are anuclear (van Herendael et al., 1978). The connections between neighboring cells in the placental amnion are marked by a complex pattern of microvilli, and the pedicels that anchor the cells into the basement membrane are more pronounced compared to the reflected amnion (van Herendael et al., 1978). Numerous perinuclear vacuoles, containing lipid substances, were found in both placental and reflected amnion (van Herendael et al., 1978). The existence of lipid granules in the cytoplasm of amniotic membrane epithelial cells was confirmed by Lemke et al. (2018), who found higher amounts in the reflected amnion, and Centurione et al. (2018), who found the highest amount in cells of the peripheral area of the placental amnion.

Differences between these regions were also found in the mesenchymal layer, by some authors also referred to as conjunctive layer of the hAM. The number of collagen fibers decreases toward distal amnion whereas the degree of anisotropy increases. The conjunctive layer of the placental amnion is richer in collagen compared to the reflected amnion (Grémare et al., 2019). Within the reflected amnion, areas near the placenta (proximal amnion) show more collagenous fibers compared to distal amnion (Connon et al., 2007). In addition, collagen fibers distal to the placental area show perpendicular alignment to the amniotic surface (Chowdhury et al., 2014), and a high degree of anisotropic arrangement (Connon et al., 2007). In contrast, collagen fibers in amnion proximal to the placenta show parallel alignment to the amniotic surface (Chowdhury et al., 2014), and a low degree of anisotropic arrangement (Connon et al., 2007), with isotropy in the placental amnion (Grémare et al., 2019).

A particular area within the reflected region has been termed zone of extreme altered morphology (Malak and Bell, 1994) or zone of weakness or weak zone (McLaren et al., 1999) due to its morphological properties and structural weakness (Malak and Bell, 1994; McLaren et al., 1999). This sub-region of the reflected amnion is located at the lower uterine pole and cervix and is sometimes also termed cervical membrane (McLaren et al., 1999) or apical amnion (Gicquel et al., 2009) [Figure 1A (2c)]. Other terms found in literature describing parts of the reflected amnion are mid-zone [Figure 1A (2b)], indicating the area halfway between the cervical area and the edge of the placental disk, and the peri-placental zone [Figure 1A (2a)] which is close to the edge of the placental disk (Malak and Bell, 1994). The peri-placental zone is also termed proximal amnion in comparison to the distal amnion [Figure 1A (2c)] which is distal to the placental disk (Connon et al., 2007).

Furthermore, in some studies the placental amnion was sub-divided into an area surrounding the umbilical cord (R1, central area) [Figure 1A (1a)] and an intermediate area (R2) [Figure 1A (1b)] between R1 and R3, where R3 (peripheral area) [Figure 1A (1c)] is close to the edge of the placental amnion (Centurione et al., 2018; Passaretta et al., 2020).

Based on the different morphological properties of the amniotic sub-regions, van Herendael concluded that the hAM epithelium has specifically adapted to perform specialized functions such as secretion, covering, and intercellular and transcellular transport (van Herendael et al., 1978). Together with the morphological observations of the conjunctive layer of the hAM, these data suggest that different amniotic sub-regions may show different effects when used for tissue regeneration.

## Stem Cell Characteristics

Stem cells represent an important factor for regenerative medicine due to their self- renewal-, proliferation-, and differentiation capacity. Numerous studies have shown that the hAM contains cells that display stem cell characteristics. Moreover, cells of the hAM have been reported to express pluripotency markers (Miki et al., 2007). For the application of (stem) cells in regenerative medicine, characterization *via* their cell marker expression profile is important.

### Stem Cell Marker Expression

The distribution of several stem cell markers in hAM samples was studied in different areas of the hAM according to their position in relation to the umbilical cord (Centurione et al., 2018). Regarding markers actually expressed in isolated hAECs, no differences were found between the sub-regions with epithelial markers (CD324, CD326, and CD73), embryonic markers [stage-specific embryonic antigen (SSEA)-4 and T cell receptor alpha locus (TRA)-1-60] and HLA-ABC class I histocompatibility antigen (Centurione et al., 2018). The same applies for markers found low/absent in expression (mesenchymal markers CD90, CD105; pericyte-associated markers CD146, CD140b, and CD49a integrin, hematopoietic marker CD45) (Centurione et al., 2018). In hAM tissue samples, the peripheral area (which is the part of the placental area closest to the reflected area), and the reflected area show the highest expression of the pluripotency markers octamer-binding transcription factor (OCT)-4 and (sex determining region Y)-box (SOX)-2 (Centurione et al., 2018). In isolated hAECs, contradictory data exist regarding the expression of OCT-4. One group found the placental hAECs to have a tendency toward higher OCT-4 expression compared to reflected hAECs (Lemke et al., 2017), while another group found no differences between reflected, placental, and umbilical hAECs for OCT-4, SOX-2, and NANOG expression (García-López et al., 2019). Although different cellular sub-populations were detected within one region by means of immunostaining (only in the nucleus/only in the cytoplasm/in both compartments), there were no differences in these sub-populations between the regions (García-López et al., 2019). In hAMSCs both regions were negative for OCT-4. However, there was a trend toward higher SSEA-4 expression in placental hAMSCs (Lemke et al., 2017). The reason for the contradictory data could result from differences in preparation, isolation and cultivation methods, and density of the cells. Since there is evidence for some cell heterogeneity between the amniotic sub-regions (Centurione et al., 2018), the question is whether this affects the proliferation and differentiation capacity of the hAM cells.

### Proliferation Capacity

Interestingly, the peripheral area of hAM tissue samples showed the highest expression level of cyclic AMP response element binding (CREB) protein. As the CREB protein has been shown to play an important part in cell differentiation and proliferation, the authors concluded that this area of the placental region has a higher proliferation and differentiation capacity (Centurione et al., 2018). A higher proliferation capacity of hAECs of the placental region compared to the reflected region was also shown in a previous study (Yoon et al., 2014). In this context, it should be mentioned that mitochondrial function (oxidative phosphorylation) controls proliferation and early differentiation potential of embryonic stem cells *in vitro* (Mandal et al., 2011). In line with this, it has been shown that hAM cells of the placental region have a higher mitochondrial oxidative phosphorylation rate compared to cells of the reflected region. This has been shown in hAM biopsies (Banerjee et al., 2015) as well as in isolated hAMSCs and hAECs (Banerjee et al., 2018a), also suggesting a higher proliferation capacity of cells of the placental region.

### Differentiation Capacity

Results from these mitochondrial respiration rate studies are also interesting in the context of the differentiation potential of hAM cells of different sub-regions. In bone marrow stromal cells, oxidative phosphorylation supports osteogenic differentiation, and its inhibition impairs this process (Shares et al., 2018). Consequently, cells of the placental region should also have a higher differentiation potential toward this lineage. Indeed, several studies provide data indicating a higher differentiation potential of cells of the placental region. The osteogenic differentiation capacity of isolated hAECs *in vitro* was examined by Centurione and colleagues. The authors performed mineralization assays and concluded that the intermediate area of the placental hAM has the highest osteogenic potential compared to all other areas (Centurione et al., 2018).

Several publications showed that placental amnion tissue expresses more parathyroid hormone-related protein (PTHrP) (Germain et al., 1992; Curtis et al., 1997; Han et al., 2008), and that placental amnion tissue (Farrugia et al., 2000) and hAECs (Germain et al., 1992) *in vitro* release more PTHrP than reflected amnion. This is interesting as it has been shown that the addition of PTHrP to human bone marrow derived mesenchymal stromal cells (Kim et al., 2008; Lee and Im, 2012), and human adipose-derived stem cells (Kim et al., 2008) has a beneficial effect on the chondrogenic differentiation capacity of the cells. The addition of PTHrP not only enhanced chondrogenesis *in vitro*, it also suppressed hypertrophy, a major limitation factor during chondrogenesis. These data indicate that the placental amnion may be more suited for stable differentiation toward cartilage.

Similar data are available for smooth muscle cell differentiation capacity. A recent publication emphasized the importance of miR-143 on the induction of the contractile phenotype in human bone marrow derived mesenchymal stromal cells differentiated toward smooth muscle cells (Yeh et al., 2019). The inhibition of miR-143 decreased contractile forces whereas overexpression increased contractile forces in the cells (Yeh et al., 2019). Kim et al. (2011) investigated miR-143 and miR-145 expression in placental and reflected amnion tissue, and found higher levels of both miRNAs in placental amnion compared to reflected amnion. This could mean that on one hand cells of the placental amnion are more likely to differentiate toward smooth muscle cells. On the other hand, other factors such as extracellular vesicles of placental amnion could also more likely stimulate cell differentiation in this direction.

The hepatic differentiation capacity of different hAM sub-regions was examined by another group (Centurione et al., 2018). They found high levels of alpha-fetoprotein in the central area and the peripheral area of hAM tissue, which could point to a higher hepatic differentiation capacity of this area (Centurione et al., 2018). However, in isolated hAECs upon hepatic differentiation, higher levels of albumin and hepatocyte nuclear factor 4α in the reflected amnion, and higher cytochrome p450 in the central area were found (Passaretta et al., 2020). These results lead to the speculation that hAECs from the central and the reflected area have a higher hepatic differentiation capacity (Passaretta et al., 2020).

A number of studies have shown that the hAM and its cells can be differentiated toward cell lineages of all three germ layers. However, it is very likely that consideration of quantitative and qualitative differences of cellular factors of the amniotic sub-regions would increase the success rate of differentiation and proliferation.

## Mechanical Properties

Beside cellular factors, mechanical properties of the cellular micro-environment such as matrix elasticity and stiffness impact cell differentiation and cell fate [reviewed in Vogel and Sheetz (2006) and Discher et al. (2009)]. In this context, it has been shown that different mechanical properties of the amniotic sub-regions strongly influence the level of corneal stem cell differentiation when the hAM is used as a substrate for cell cultivation (Chen et al., 2012). The results of this study confirmed that there is a correlation between the level of stem cell differentiation and the level of tissue stiffness (Chen et al., 2012). Compared to proximal hAM, samples taken from distal to the placental disk showed a greater stiffness, and at the same time a higher expression of cytokeratin, which is a marker for terminally differentiated corneal epithelial cells (Chen et al., 2012). Considering the hypothesis that cells for the treatment of ocular surface stem cell disorder should be in a rather undifferentiated state, the authors recommended to use the hAM of the area proximal to the placental disk (Chen et al., 2012). Of note, in regard to the therapeutic use of human mesenchymal stem cells, it has been shown that appropriate *in vitro* matrix conditions could support the cells to overcome inappropriate *in vivo* conditions (Engler et al., 2006).

In addition to differences in stiffness between amniotic regions, differences in strength (Massie et al., 2015; Grémare et al., 2019) have also been noted. On studying intra-donor variability, Grémare et al. (2019) found placental hAM to be stronger (maximal force) and also more stretchable (strain at break) than reflected hAM. As the mechanical properties of hAM appear to be critical for its application, for example, as biological conduit for vascular tissue engineering (Amensag and McFetridge, 2012; Peirovi et al., 2012), the sub-regional differences in strength should be taken into account. In this regard, attention should also be paid to the zone of altered morphology (Malak and Bell, 1994). This area showed only half of the rupture strength compared to the remaining reflected amnion (El Khwad et al., 2005), making it an unsuitable material for tissue engineering where mechanical properties are important.

Although seen as proper material for graft construction, the hAM has also been criticized for its poor tear resistance under certain circumstances (Muralidharan et al., 1991; Farhadihosseinabadi et al., 2018). It is reasonable to speculate that the outcome could be improved when selecting the hAM from the placental region for graft construction.

## Bioactive Factors for Tissue Regeneration

A key part of the beneficial effects of hAM in tissue regeneration can be attributed to its bioactive factors, modulating several cellular processes. For tissue regeneration, modulation of the inflammatory process is a prerequisite, determining the quality of the regenerative response. In this context, the wound healing properties of the hAM have been partially attributed to its anti-inflammatory transforming growth factor beta (TGFB) content (Han et al., 2008). Interestingly, TGFB1/TGFB2/TGFB3 and transforming growth factor beta receptor (TGFBR)1 and TGFBR2 showed a higher protein expression in reflected amnion compared to placental amnion tissue (Han et al., 2008). In addition, the histocompatibility antigen class I-G (HLA-G) a protein, which induces apoptosis of CD8b T cells and fosters T helper cell type (Th)2 response, showed higher mRNA expression in reflected amnion than in placental amnion (Han et al., 2008). In line with this, analysis of conditioned medium from reflected hAM explants showed a trend toward higher concentrations of the immune-modulatory surfactant protein D (Lemke et al., 2017). However, the immune-modulatory surfactant protein A was predominantly found in the placental amnion tissue (Lee et al., 2010a). In sum, reflected hAM seems to have more immune-modulatory potential than placental hAM.

In contrast, the placental region seems to be more pro-inflammatory. This can be seen by the higher expression of proteins, mostly known for their pro-inflammatory nature, such as C-X-C motif chemokine 6 (Han et al., 2008), and prostaglandin-endoperoxide synthase 2 (PTGS2) or cyclooxygenase 2 (COX2) (Lee et al., 2010b) in placental hAM tissue. Furthermore, placental hAMSCs incubated without lipopolysaccharide (LPS) (Banerjee et al., 2018b), and placental hAM explants incubated with LPS (Han et al., 2008) showed a much stronger pro-inflammatory response in terms of interleukin (IL)-6 release (Banerjee et al., 2018b) and mitogen-activated protein kinase (MAPK) 3/MAPK1 activation and IL1B mRNA expression (Han et al., 2008) compared to reflected amnion samples. These observations are particularly interesting considering the fact that therapeutic cells are often transplanted in a microenvironment with a high degree of inflammatory factors. Besides, some inflammatory cytokines such as IL-6 (Mauer et al., 2014) not only propagate the immune response but also promote the regenerative process by preventing cell death, enhancing cell motility and migration, and stimulating growth factor expression [reviewed in Karin and Clevers (2016)].

The hAM contains several growth factors that have beneficial effects on wound healing processes (Rousselle et al., 2019). Beside the aforementioned TGFB family (Han et al., 2008), higher protein levels of epidermal growth factor could also be detected in reflected hAM tissue (mid-zone and apical amnion) compared to placental hAM (Gicquel et al., 2009). In contrast, other growth factors which are important for keratinocyte migration (Seeger and Paller, 2015), such as keratinocyte growth factor (Han et al., 2008), insulin-like growth factor 1 (Han et al., 2008), insulin-like growth factor-binding protein (Litwiniuk et al., 2017), and hepatocyte growth factor (Banerjee et al., 2018b) were higher in placental compared to reflected amnion. In addition, PTHrP, a hormone which enhances differentiation of keratinocytes (Kaiser et al., 1994) and promotes fracture healing [reviewed in Kostenuik and Mirza (2017)], showed higher protein expression levels (Germain et al., 1992; Curtis et al., 1997; Han et al., 2008) and higher *in vitro* secretion (Germain et al., 1992; Farrugia et al., 2000) in placental amnion compared to reflected amnion. Wound healing is known to be a complex matter. Therefore, the knowledge on specific factors could facilitate the decision on which region to select for a specific application. However, a number of factors play a dual role. For example, TGFB is an important factor for the promotion of wound healing (Han et al., 2008). However, its potential pro-fibrotic function (Leask and Abraham, 2004; Walton et al., 2017) should be taken into account if the prevention of scar tissue formation is important. For example, for nerve regeneration, scar formation can impair regeneration at the sites of peripheral nerve repair (Atkins et al., 2006).

Regarding factors regulating angiogenesis, the placental region tends to be more pro-angiogenic. Angiogenin (Litwiniuk et al., 2017) and olfactomedin-like protein 3 (Han et al., 2008) were higher in placental amnion tissue compared to reflected amnion. Both proteins are known to induce angiogenesis (Kishimoto et al., 2005; Miljkovic-Licina et al., 2012). In addition, PTGS2 or COX2, which plays an important part in inflammatory cytokine-induced angiogenesis (Kuwano et al., 2004), was primarily localized in placental amnion tissue but not in reflected amnion (Lee et al., 2010b). On the other hand, reflected amnion tissue, especially the cervical region, contains very low levels of pro-angiogenic factors (Litwiniuk et al., 2017). This has been suggested to be possibly beneficial for some ophthalmological applications to avoid induction of angiogenesis (Litwiniuk et al., 2017).

Beside the management of angiogenesis, for ophthalmological applications, one has to consider the formulation of the hAM and the mode of application. If the hAM is applied permanently in its intact form (with the epithelial layer), for ocular reconstruction where optical properties play a role, it seems to be advantageous to use distal (reflected) hAM. Compared to proximal (reflected) and placental hAM, distal (reflected) hAM is more transparent (Connon et al., 2010; Massie et al., 2015; Deihim et al., 2016). Of note, the presence of epithelial cells seems to be the driving factor for the transparency differences between the amniotic regions (Deihim et al., 2016). The hAM can also be used as “therapeutic contact lens” for the treatment of epithelial cornea defects (Gris et al., 2002). With this method, the hAM temporarily covers the lesion thereby protecting the cornea surface from the mechanical trauma of blinking, and induces epithelialization (Gris et al., 2002). As discussed before, the placental region is rich in growth factors which favor epithelialization by stimulation of keratinocyte migration. Therefore, the placental hAM seems to be more appropriate for this application. This is in line with a recent study, where the authors suggested using placental hAM for corneal surface regeneration based on molecular structure and chemical findings (Kim et al., 2014). One drawback of the placental hAM was the dense collagenous structure compared to the reflected hAM (Kim et al., 2014). However, as the hAM detaches or dissolves after a certain period of time, corneal transparency will be restored (Gris et al., 2002). Also for the treatment of corneal ulcers, Connon et al. (2010) suggested to use freeze-thawed hAM of proximal amnion as the refractive index of proximal amnion is much closer to that of the human cornea compared to distal amnion, which can positively affect the clinical outcome.

Another crucial factor for the regeneration of tissue is the presence of functional mitochondria, as they are important regulators of cell metabolism, cell survival, and cell death. Tissue regeneration is an energy consuming process, which can be enhanced by increasing mitochondrial oxidative phosphorylation (Shyh-Chang et al., 2013). Beside the generation of the energy carrier adenosine triphosphate (ATP), mitochondria have been shown to control (stem) cell fate [reviewed in Ito and Ito (2016)]. With regard to the hAM, studies show that mitochondria are involved in processes leading to the rupture of membranes [reviewed in Weidinger and Banerjee (2020)]. As the reflected region is the site of rupture, it does not come as a surprise that mitochondria of the placental and reflected region differ in morphology and functional activity (Banerjee et al., 2015, 2018a). The placental region is characterized by higher mitochondrial activity and ATP levels in the intact tissue (Banerjee et al., 2015), and in isolated hAECs and hAMSCs (Banerjee et al., 2018a). These data indicate that tissue or cells of the placental region could be more suitable for certain applications such as the regeneration of metabolically active tissues such as liver or muscle. Moreover, mitochondria of cells of the placental amnion seem to have a greater potential to adapt to a challenging microenvironment (Banerjee et al., 2018a). This could be particularly important for the transplantation of amniotic cells into inflamed micro-environments. Another example on how differently cells of the amniotic sub-regions react to their micro-environment was shown with different cultivation conditions. Four days of culture of hAMSCs at 20% oxygen strongly increased the rate of mitochondrial oxidative phosphorylation in cells of the placental region but not in cells of the reflected region (Banerjee et al., 2018b). This is important to note, since, as mentioned before, the rate of oxidative phosphorylation can influence the readiness of a cell to proliferate or differentiate.

Due to its biological age, the hAM is also particularly suitable for the generation of induced pluripotent stem cells. Notably, it has been shown that there is a correlation between the metabolic pattern of a cell and the reprogramming efficiency (Panopoulos et al., 2012). Pluripotent stem cells have a low oxidative phosphorylation rate (Zhou et al., 2012). This metabolic pattern comes closer to that of the reflected amnion than that of the placental amnion. Therefore, the reprogramming efficiency and velocity could be higher in cells of the reflected amnion compared to the placental amnion.

Bioactive molecules that are closely related to mitochondria are reactive oxygen species (ROS), as mitochondria are the main ROS producers under physiological conditions. Besides mitochondria [reviewed in Andreyev et al. (2015)], enzymes such as the NADPH oxidase (NOX) contribute to ROS production [reviewed in Lambeth (2004)]. ROS can cause damage to biomolecules but are also a crucial factor for the transduction of signaling pathways [reviewed in Weidinger and Kozlov (2015)]. Measurements of intra- and extracellular ROS in hAM tissue and cells revealed higher levels of intracellular ROS in the reflected region compared to the placental region (Banerjee et al., 2015, 2018a). In contrast, extracellular ROS were higher in the placental region compared to the reflected region (Banerjee et al., 2018a). In addition, measurement of NOX activity showed higher activity in placental amnion tissue than reflected amnion (Banerjee et al., 2018a). These results are particularly interesting for several reasons. First, it has been shown that NOX-derived ROS participate in the stimulation of angiogenesis [reviewed in Ushio-Fukai and Urao (2009)]. This would support the aforementioned assumption that the placental region is more pro-angiogenic. Of note, it has been shown that aquaporines are required for growth factor-induced NOX signaling (Miller et al., 2010). In line with this, Bednar et al. (2015) observed that mRNA expression of several aquaporines was higher in placental compared to reflected amnion tissue. Second, also NOX-derived ROS from non-phagocytic cells play a role in the defense to invading microorganisms [reviewed in Grandvaux et al. (2007)]. This fact led to the speculation that the NOX activity in the hAM may contribute to its antimicrobial effect (Banerjee et al., 2018a), making the placental region more effective in this regard.

## Cultivation of hAM Cells

As a final note, it should be pointed out that due to the different properties of the different amniotic sub-regions, tissue or isolated cells can behave or respond differently in culture, which in turn can impact an experiment or clinical application. For example, isolated cells of the placental region adhere more readily to collagen-coated surfaces compared to cells of the reflected region (Banerjee et al., 2018a). Consequently, the cell densities of cells of the reflected region are lower during cultivation compared to cells of the placental region. Of note, cell confluence affects the expression of proteins (Ren et al., 2015), cell metabolism (Gal et al., 1981), and mechanical properties (Efremov et al., 2013) among others. For cultivating reflected and placental amniotic cells separately, higher numbers of reflected amniotic cells have to be seeded in order to achieve similar cell confluence as placental amniotic cells. Furthermore, mixed cultures of amniotic cells contain higher numbers of placental amniotic cells than reflected amniotic cells, which could influence the regenerative outcome upon clinical application. Moreover, it has been shown that tissue and cells of the amniotic sub-regions react differently to the same stimulus. For example, Astern et al. (2013) showed that stimulation with pre-B cell colony enhancing factor increased amnion permeability in the placental region but not in the reflected region. Another example is that, as mentioned before, mitochondrial oxidative phosphorylation can be influenced by different oxygen concentrations during cultivation (Banerjee et al., 2018b). These studies emphasize how much the cultivation conditions can influence cell fate, which in the long run also has an impact on the application outcome.

## Theoretical Explanations for Sub-Regional Differences of hAM

Providing a protective environment for the embryo/fetus is an extremely important function. In order to best fulfill its biological function, the cells in the different regions of the amnion appear to be specialized in certain functions (van Herendael et al., 1978). As an example, van Herendael cited the more pronounced intercellular channels of placental hAECs compared to reflected hAECs, probably indicating increased intercellular and transcellular transport in the placental region (van Herendael et al., 1978).

The reasons for the various differences observed could be due to the developmental characteristics of the hAM sub-regions (Han et al., 2008), or the impact of the anatomical region (Han et al., 2008; Yoon et al., 2014; Banerjee et al., 2018a, b; Poženel et al., 2019). The placental region covers the chorionic plate and the reflected region covers the chorionic leave (Germain et al., 1992). Importantly, the chorionic plate is vascular whereas the chorionic leave is avascular (Ernst, 2011). Therefore it is likely that cells of the placental region are supplied with different amounts of oxygen and nutrients at different frequencies compared to the reflected region. Unfortunately, no data exist on differences of the micro-environments of cells of reflected and placental amnion *in vivo*. However, an indication for differences of the micro-environments could be that, within the same cell type (hAECs, hAMSCs), different mtDNA copy numbers per cell were found according to the amniotic sub-regions (Banerjee et al., 2018a). A possible epigenetic regulation such as demethylation has been suggested as the cause of these differences (Banerjee et al., 2018b). In this context, it is important to know that the enzymatic activities of some demethylases can be modulated by oxygen availability and cellular metabolism (Lamadema et al., 2019).

Furthermore, it is known that different availabilities of oxygen and/or nutrients also result in different proliferation and differentiation capacities, and secretory behavior of cells. Indeed, it was shown that under different oxygen conditions *in vitro*, hAMSCs of the placental amnion secrete different amounts of IL-6 (Banerjee et al., 2018b). In addition, the fact that the placental region has possibly a higher availability of oxygen could also be the reason for the cells of the placental region to react stronger to changes in oxygen levels *in vitro* compared to cells of the reflected region (Banerjee et al., 2018b).

In our opinion, several reasons lead to sub-regional differences of amniotic cells. Most likely, the cells of the placental and reflected amnion diverge at some point of their development. As a result of their different functionality, the cells also react differently to extrinsic influences, such as oxygen and nutrients, consequently leading to different proliferation, differentiation, and secretory behavior.

## Conclusion

Taken together, many studies show that the sub-regions of the hAM differ quantitatively and qualitatively in their properties (Supplementary Table 1). A number of studies provide robust evidence that the sub-regions of the hAM differ in properties affecting cell proliferation, differentiation, immune-modulation, inflammation, and angiogenesis. Furthermore, the hAM also shows different physical properties such as tear resistance and transparency according to the amniotic sub-region.

Although data of these studies have raised more questions that have to be addressed in future studies, by now the sub-regional differences found in the hAM have established a consensus among researchers. However, despite the evidence provided by research, the conclusions drawn have not been translated to clinical application so far. In the clinics, the hAM continues to be applied unselected, as one homogeneous tissue. Yet, there are several indications that the differences found so far could influence the outcome of a clinical application when the hAM is used as a source for cells, substrates, scaffolds or bioactive factors. Therefore, with this review, we aim to address researchers in translational settings and clinicians to re-evaluate the application of the hAM for therapeutic use, by including the current state of knowledge as selection criterion.

We strongly recommend considering the hAM sub-regions for the design of future studies. We are aware that this will not always be an easy task. On one hand, some regions are much smaller than others, meaning that one may have to pool several donors. On the other hand, it is often difficult to detect a certain region. For example, the umbilical cord is rarely localized in the center of the placenta, and it is therefore difficult to divide the placental region into sub-regions. However, consideration of region-specific properties will most certainly help to optimize and fine-tune the clinical application of the hAM.

## Author Contributions

AW and AB conceptualized the manuscript. AW, LP, and AB drafted the manuscript and figures. AW, LP, SW, and AB critically read/edited the manuscript. All authors approved the manuscript.

## Conflict of Interest

The authors declare that the research was conducted in the absence of any commercial or financial relationships that could be construed as a potential conflict of interest.

## Abbreviations

AMP: adenosine monophosphate
ATP: adenosine triphosphate
COX: cyclooxygenase
CREB: cyclic AMP response element binding
hAEC: human amniotic membrane epithelial cells
hAM: human amniotic membrane
hAMSC: human amniotic membrane mesenchymal stromal cells
HLA: human leukocyte antigen
IL: interleukin
LPS: lipopolysaccharide
MAPK: mitogen-activated protein kinase
NADPH: nicotinamide adenine dinucleotide phosphate
NOX: NADPH oxidase
OCT-4: octamer-binding transcription factor 4
PTGS: prostaglandin-endoperoxide synthase
PTHrP: parathyroid hormone-related protein
ROS: reactive oxygen species
SOX-2: (sex determining region Y)-box 2
SSEA-4: stage-specific embryonic antigen 4
TGFB: transforming growth factor beta
TGFBR: transforming growth factor beta receptor
Th2: T helper cell type 2
TRA-1-60: T cell receptor alpha locus.
