# Supplementary material for: Sub-Regional Differences of the Human Amniotic Membrane and Their Potential Impact on Tissue Regeneration Application
Source: Front Bioeng Biotechnol. 2021 Jan 13;8:613804. doi: 10.3389/fbioe.2020.613804 (PMC7839410; doi:10.3389/fbioe.2020.613804)
Supplement: Supplementary file 1 [file Table_1.pdf]

Tissue/cells	Parameter	Regional differences	References	Potential impact on	
				suggested*	proven
Stem cell marker					
hAECs	epithelial markers (CD324, CD326, CD73)	no	Centurione *et al.* 2018	-	-
hAECs	embryonic markers (SSEA-4, TRA-1-60, HLA-ABC)	no	Centurione *et al.* 2018	-	-
hAECs	CD90, CD105, CD146, CD140b, CD49a integrin, CD45	no	Centurione *et al.* 2018	-	-
hAECs	OCT-4	yes/no	Lemke *et al.* 2017, García-López *et al.* 2019	-	-
hAMSCs	OCT-4	no	García-López *et al.* 2019	-	-
hAMSCs	SSEA-4	yes	Lemke *et al.* 2017	-	-
tissue	OCT-4, SOX-2	yes	Centurione *et al.* 2018	-	-
Proliferation and differentiation capacity					
hAECs	proliferation capacity	yes	Yoon *et al.* 2014	-	proliferation capacity (Yoon *et al.* 2014)
hAECs	osteogenic potential	yes	Centurione *et al.* 2018	-	-
hAECs	albumin, hepatocyte nuclear factor 4alpha	yes	Passaretta *et al.* 2020	hepatic differentiation capacity (Passaretta *et al.* 2020)	-
tissue and cells	PTHrP expression and release	yes	Germain *et al.*1992, Curtis *et al.* 1997, Farrugia *et al.* 2000, Han *et al.* 2008	chondrogenic differentiation capacity<br>differentiation of keratinocytes<br>wound healing processes	-
tissue	miR-143, miR-145	yes	Kim *et al.* 2011	smooth muscle cell differentiation capacity	-
tissue	alpha-fetoprotein	yes	Centurione *et al.* 2018	hepatic differentiation capacity (Centurione *et al.* 2018)	-
tissue	CREB protein	yes	Centurione *et al.* 2018	proliferation capacity<br>differentiation capacity<br>(Centurione *et al.* 2018)	-
Physical properties					
tissue	stiffness	yes	Chen *et al.* 2012	-	corneal stem cell differentiation (Chen *et al.* 2012)
tissue	strength	yes	Gremare *et al.* 2019, Massie *et al.* 2015	suitability as graft for vascular tissue engineering	-
tissue	transparency	yes	Connon *et al.* 2010, Deihim *et al.* 2016, Massie *et al.* 2015, Kim *et al.* 2014	corneal transparency post-application	-

Tissue/cells	Parameter	Regional differences	References	Potential impact on	
				suggested*	proven
Immune-active factors					
hAMSCs	IL-6 release	yes	Banerjee *et al.* 2018b	pro-inflammatory potential	-
hAM explants	IL1B mRNA, MAPK3/MAPK1 activation	yes	Han *et al.* 2008	pro-inflammatory potential	-
hAM explants	surfactant protein D	yes	Lemke *et al.* 2017	immune-modulatory potential	-
tissue	TGFB content	yes	Han *et al.* 2008	immune-modulatory potential<br>wound healing capacity	-
tissue	HLA-G mRNA	yes	Han *et al.* 2008	immune-modulatory potential<br>(Han *et al.* 2008)	-
tissue	surfactant protein A	yes	Lee *et al.*2010a	immune-modulatory potential<br>(Lee *et al.*2010b)	-
tissue	C-X-C motif chemokine 6, PTGS2 (COX2)	yes	Han *et al.* 2008, Lee *et al.* 2010b	pro-inflammatory potential	-
Factors influencing wound healing and angiogenesis					
tissue, hAMSCs	KGF, insulin-like growth factor 1, insulin-like growth factor binding protein, HGF	yes	Han *et al.* 2008, Litwiniuk *et al.* 2017,<br>Banerjee *et al.* 2018b	keratinocyte migration<br>wound healing	-
tissue	EGF	yes	Gicquel *et al.* 2009	epithelial wound healing	-
tissue	angiogenin, olfactomedin-like protein 3	yes	Han *et al.* 2008, Litwiniuk *et al.* 2017	angiogenesis	-
tissue	PTGS2 (COX2)	yes	Lee *et al.* 2010b	angiogenesis	-
Mitochondria and reactive oxygen species					
tissue, cells	mitochondrial activity	yes	Banerjee *et al.* 2015, 2018a	regeneration of metabolically active tissue<br>potential to adapt to injured/inflamed environment (Banerjee *et al.*2018a)<br>reprogramming efficiency	-
tissue, cells	mitochondrial oxidative phosphorylation	yes	Banerjee *et al.* 2015, 2018a	proliferation capacity<br>differentiation capacity	-
tissue, cells	intra- and extracellular ROS	yes	Banerjee *et al.* 2015, 2018a	signaling function	-
tissue	NOX activity	yes	Banerjee *et al.* 2018a	stimulation of angiogenesis<br>antimicrobial property<br>(Banerjee *et al.*2018a)	-
tissue	aquaporine mRNA	yes	Bednar *et al.* 2015	NOX signaling	-

Table 1. Potential impact of cell- and tissue-specific properties of the human amniotic membrane on tissue regeneration applications with regard to amniotic sub-regions. * References for the evaluation of the potential impact are provided in brackets. If no reference is provided, the evaluation of the impact is suggested by the authors of the review based on the data provided in the respective publication. Abbreviations: COX, cyclooxygenase; CREB, cyclic AMP response element binding; EGF, epidermal growth factor; hAECs, human amniotic membrane epithelial cells; hAMSCs, human amniotic membrane mesenchymal stromal cells; HLA, human leukocyte antigen; HGF, hepatocyte growth factor; IL, interleukin; KGF, keratinocyte growth factor, MAPK, mitogen-activated protein kinase; NOX, nicotinamide adenine dinucleotide phosphate oxidase; OCT-4, octamer-binding transcription factor 4; PTGS, prostaglandin-endoperoxide synthase; PTHrP, parathyroid hormone-related protein; ROS, reactive oxygen species; SOX-2, (sex determining region Y)-box 2; SSEA-4, stage-specific embryonic antigen 4; TGFB, transforming growth factor beta; TRA, T cell receptor alpha locus.
